# An estimation of global *Aeromonas* infection prevalence in children with diarrhoea: a systematic review and meta-analysis

**DOI:** 10.1186/s12887-023-04081-3

**Published:** 2023-05-22

**Authors:** Hamid Sadeghi, Ahad Alizadeh, Majid Vafaie, Mohammad Reza Maleki, Saeideh Gholamzadeh Khoei

**Affiliations:** 1 Medical Microbiology Research Center, Qazvin University of Medical Sciences, Qazvin, Iran; 2 Metabolic Diseases Research Center, Research Institute for Prevention of Non-Communicable Diseases, Qazvin University of Medical Sciences, Qazvin, Iran; 3 Clinical Research Development Unit, Qods Hospital, Qazvin University of Medical Sciences, Qazvin, Iran; 4 Clinical Research Development Unit, Kowsar Hospital, Qazvin University of Medical Sciences, Qazvin, Iran

**Keywords:** *Aeromonas*, Diarrhoea, Children, Global, Systematic review, Meta-analysis

## Abstract

**Objectives:**

Diarrhoea is the most commonly related disease caused by *Aeromonas*. To improve knowledge on prevalence, this systematic review and meta-analysis was performed to evaluate the global prevalence of *Aeromonas* in children with diarrhoea worldwide.

**Methods:**

We systematically searched PubMed, Google scholar, Wiley Online Library, ScienceDirect, and Web of sciences to identify all cross-sectional published papers between 2000 and 10 July 2022. After initial scrutinizing, 31 papers reporting the prevalence of *Aeromonas* in children with diarrhoea were found to be adequate for meta-analysis. The statistical study was accompanied by using random effects models.

**Results:**

A total of 5660 identified papers, 31 cross-sectional studies encompassing 38,663 participants were included in the meta-analysis. The pooled prevalence of *Aeromonas* in children with diarrhoea worldwide was 4.2% (95% CI 3.1–5.6%). In the subgroup analysis, the highest prevalence was seen among children in Upper middle-income countries with pooled prevalence of 5.1% (95% CI 2.8–9.2%). The prevalence of *Aeromonas* in children with diarrhoea was higher in countries with populations of over 100 million people (9.4%; 95% CI 5.6–15.3%), and water and sanitation quality score of less than 25% (8.8%; 95% CI 5.2–14.4%). Additionally, Cumulative Forest Plot showed a decreasing trend in the prevalence of *Aeromonas* infection in children with diarrhoea over time (*P* = 0.0001).

**Conclusion:**

The results of this study showed a better comprehension of *Aeromonas* prevalence in children with diarrhoea on a global scale. As well as our findings showed that much work is still required to decline the burden of bacterial diarrhoea in countries with high populations, low-level income, and unsanitary water.

## Introduction

Diarrhoeal diseases remain a serious worldwide problem among young children. In 2016, diarrhoea was the fifth main cause of fatality among children younger than 5 years, approximately 27% of diarrhoeal deaths occurred among children [1]. Some causes of diarrhoea involve infection by bacteria, viruses, parasites, and other non-infectious causes [2]. Bacteria are responsible for 20–40% of diarrhoea diseases, and various bacterial pathogens have been mostly attributed to diarrhoea episodes, including *Escherichia coli*, *Campylobacter jejuni*, *Salmonella spp*., *Yersinia enterocolitica*, *Vibrio cholerae*, *Plesiomonas spp*, and *Aeromonas* spp [3]. The rank of *Aeromonas* vary from first [4] to fifth [5] among enteropathogenic bacteria that cause diarrhoea. Childhood diarrhoea is most often caused by *Aeromonas* species [6] and in bacteremia disseminated from gastrointestinal tract have mortality rates of 30–70% [7]. Diarrhoea is the common manifestation of *Aeromonas* infection. Aeromonas has also been related to a variety of extraintestinal presentations. [8]. *Aeromonas*-associated diarrhoeal is defined as the leading cause of mortality with 1.0 cases per 100,000 [1]. Symptoms of *Aeromonas* related diarrhoea are quietly changeable, consistency of stool varied from watery to loose to bloody; and diarrhoea is either self-limited, tolerable to one week, or elongated up to two weeks, or become chronic with more than one month period [4, 9]. It is noteworthy that 2,195 children die due to diarrhoea every day, more than malaria, AIDS, and measles combined worldwide [10]. Several investigations have been conducted in many parts of the world at several times to record the prevalence of *Aeromonas* genus. Anyway, briefed prevalence information of this bacterial disease in diarrhoea children worldwide is needed. Accordingly, the existing study is the first of its kind to specify the pooled prevalence of *Aeromons* in diarrhoea children on a global scale.

## Methods

### Search strategy

This systematic review and meta-analysis followed PRISMA guidelines (http://www.prisma-statement.org/). A comprehensive literature search was carried out to estimate the prevalence of *Aeromonas* in children with diarrhoea. Papers were identified using a literature search in five English-language databases (PubMed, Google scholar, Wiley Online Library, ScienceDirect, and Web of sciences) between 2000 and 10 July 2022 using the following keywords: “*Aeromonas*” “Children” “diarrhoea” alone or in combination with “OR” and/or “AND” operators. Published studies with focused on the epidemiology were selected. All records were imported in Endnote version X8 (Clarivate Analytics, Philadelphia, PA, USA). The limits of language and study group were English and children respectively.

### Eligibility criteria

We skimmed titles and abstracts of studies based on determined inclusion and exclusion criteria. The following five inclusion criteria were selected in the current systematic review and meta-analysis: (A) original available full text research papers; (B) studies design been cross-sectional; (C) All papers related to the prevalence of *Aeromonas* in children with diarrhoea; (D) literatures published in the English language; and (E) Published papers between 2000 and 10 July 2022 were considered. The exclusion criteria were empirical investigations, review papers, clinical trials, letters to the editor, case report articles, unpublished studies, confusing studies, meeting abstracts, congress, case control, cohort studies, and short communication articles.

### Data extraction

The data extraction was carried out by 2 members of the research team (HS and SGK) independently from included studies using a pretested format prepared in Microsoft Excel. Following a careful study of fulltext papers, information such as the name of first author, country, year of publication, patients, sample size, type of sample, type of study, prevalence of *Aeromonas*, and positive samples were exteracted. Any disagreements between the two research team members were resolved by discussion and meeting with a third research team member (AA).

### Quality assessment

The Newcastle–Ottawa scale was used for assessing the quality of included articles [11]. A score with a most of 10 points was given to each paper according to subject selection (0–5 points), comparability of subjects (0–2 points), and outcome (0–3 points). A total score of 0–4, 5–6, 7–8, and 9–10 points was addressed Unsatisfactory Studies, Satisfactory Studies, Good Studies, and Very Good Studies, respectively [12].

### Data synthesis and statistical analysis

All statistical analysis were carried out using Comprehensive Meta-Analysis (version 3) software. The original papers were explained using forest plot, tables, and figures. Whereof there was heterogeneity among surveys, random effect model was used to evaluate the pooled prevalence. The pooled prevalence of *Aeromonas* in children with diarrhoea did report globally and was estimated with 95% confidence intervals (CIs). Sub-group analysis included country income level, population, and water and sanitation quality score. The possibility of publication bias was studied using Egger’s regression test and Begg’s test. A meta-regression analysis was carried out to assess the effect of the year of publication on prevalence. Cochrane’s Q test and heterogeneity index (I^2^ statistics) were used to calculate the amount of heterogeneity among included papers, with I^2^ values of 25%, 50%, and 75% known as low, moderate, and high heterogeneity, respectively [13]. A *P* value < 0.05 was considered statistically significant.

## Results

### Details of prevalence publications

**Fig. 1:**
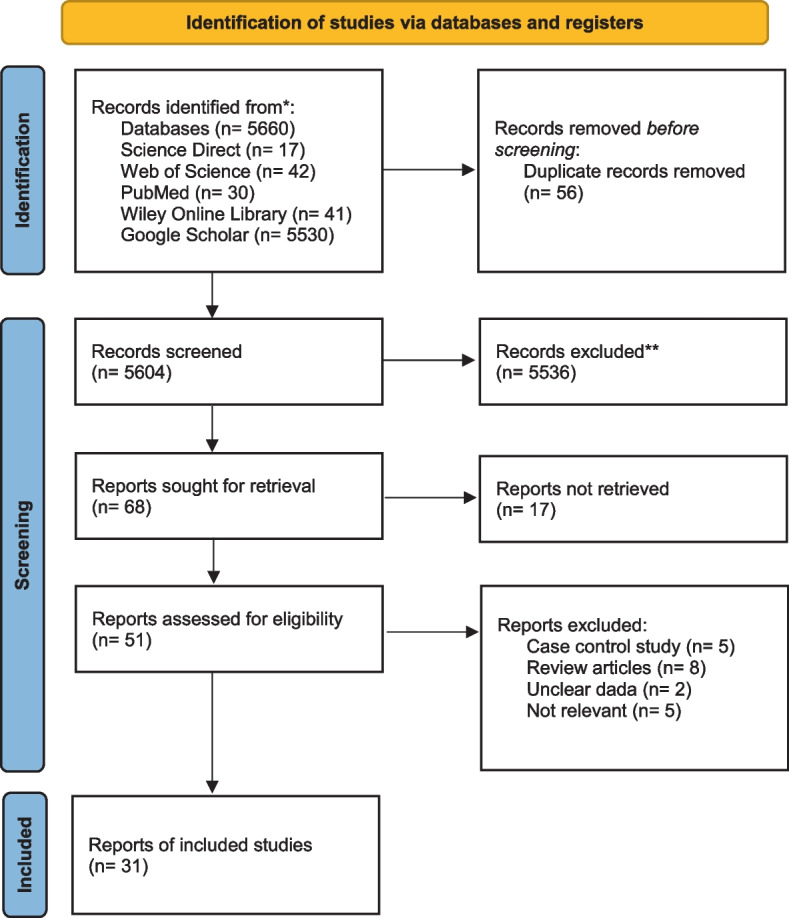
Schematic diagram showing the literature search with exclusion/inclusion procedure for meta-analysis

Through online database search, we returned a total of 5660 papers. Review papers on the prevalence of *Aeromonas* in children with diarrhoea were removed. After the primary check of the titles of eligible papers, related articles to the prevalence of *Aeromonas* were selected, while irrelevant papers were excluded from the study. Finally, thirty one papers were included in the meta-analysis (Fig. 1) [6, 14–43].

### Meta-analysis of *Aeromonas* prevalence in diarrhoea children

**Fig. 2:**
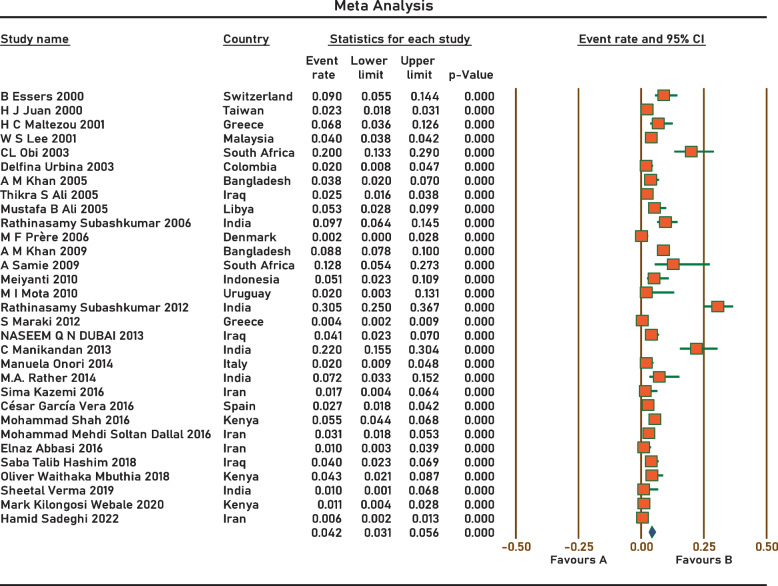
Forest plots for random-effects meta-analysis of the prevalence of *Aeromonas*

**Fig. 3:**
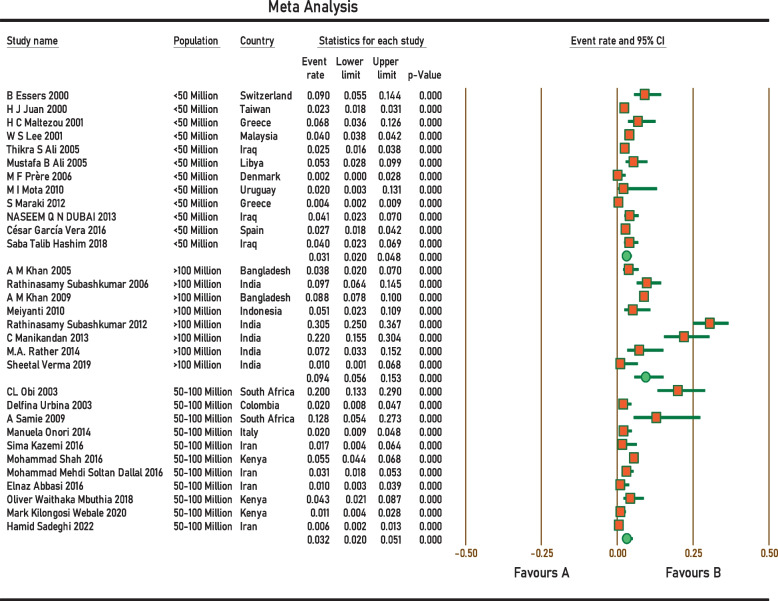
Sub-group analysis of the prevalence of *Aeromonas* in included studies based on population

**Fig. 4:**
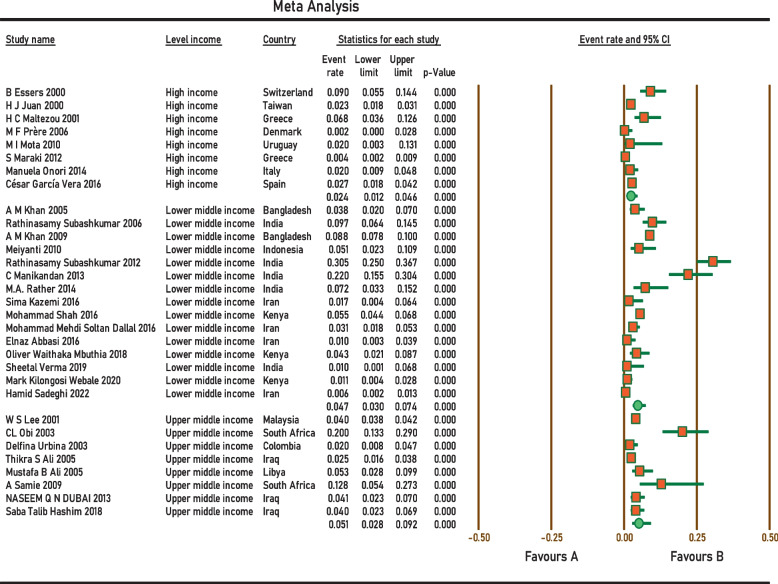
Sub-group analysis of the prevalence of *Aeromonas* in included studies based on income level

**Fig. 5:**
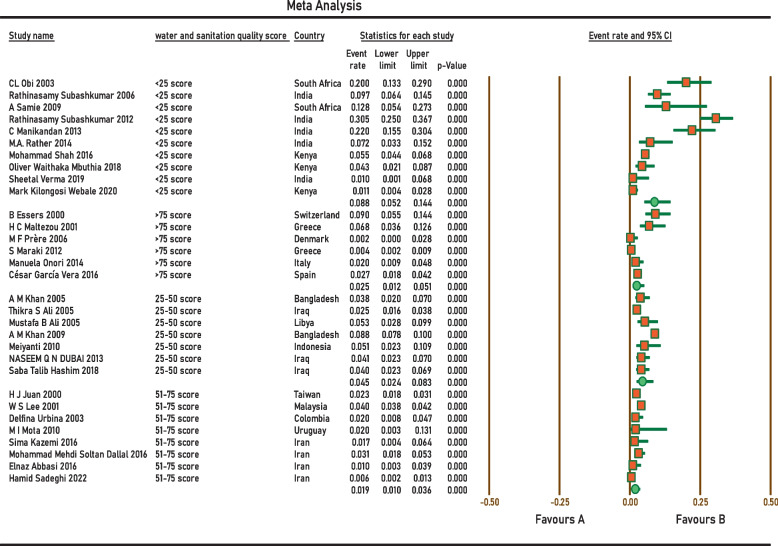
Sub-group analysis of the prevalence of *Aeromonas* in included studies based on water and sanitation quality score

**Fig. 6:**
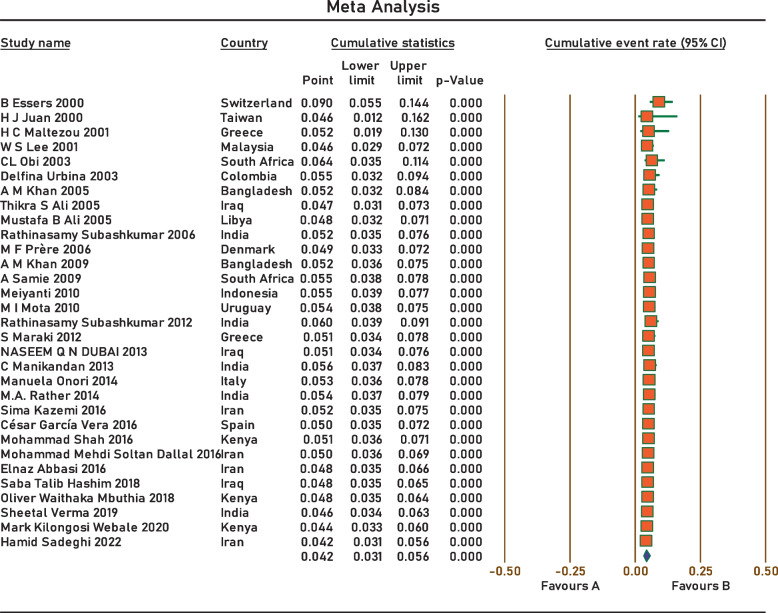
Forest plots for random-effects meta-analysis of the prevalence of *Aeromonas* during the time

Current survey covered four continents: Europe, Asia, Africa, and South America. No related published papers were found for North, Central America, and Australia. The number of papers included in the meta-analysis was 31, including 38,663 samples, from 2000 to 2022. The principal specifications and outcomes of the included studies are presented in Table 1. Included studies showed high heterogeneity (I^2^ = 95.27%; *p* < 0.0001), which is indicative to use random effects model. The pooled prevalence by the random-effects model was 4.2% (95% CI 3.1–5.6%) (Fig. 2). Subgroup analysis showed that regions with populations of over 100 million people had the highest prevalence of *Aeromonas* (9.4%; 95% CI 5.6–15.3%) (Fig. 3). In addition, the infection was more prevalent in Upper middle-income regions 5.1% (95% CI 2.8–9.2%) (Fig. 4), and regions with water and sanitation quality score of less than 25% (8.8%; 95% CI 5.2–14.4%) (Fig. 5). Our results identify evidence of decreasing *Aeromonas* infection in children with diarrhoea within the approximately more than two decades covered by reported studies (Fig. 6).

**Table 1:** Characteristics of the included studies

No	Study	Country	Year of publication	Patients	Sample size	Type of sample	Type of study	Prevalence	Positive samples	Quality score
1	B Essers	Switzerland	2000	children	166	diarrheal stool	cross-sectional	9.0%	15	6
2	H J Juan	Taiwan	2000	children	2150	diarrheal stool	cross-sectional	2.3%	50	1
3	H C Maltezou	Greece	2001	children	132	diarrheal stool	cross-sectional	6.8%	9	4
4	W S Lee	Malaysia	2001	children	26,444	diarrheal stool	cross-sectional	4.0%	1057	4
5	CL Obi	South Africa	2003	children	100	diarrheal stool	cross-sectional	20.0%	20	7
6	Delfina Urbina	Colombia	2003	children	253	diarrheal stool	cross-sectional	2.0%	5	7
7	A M Khan	Bangladesh	2005	children	240	diarrheal stool	cross-sectional	3.75%	9	6
8	Thikra S Ali	Iraq	2005	children	850	diarrheal stool	cross-sectional	2.47%	21	4
9	Mustafa B Ali	Libya	2005	children	169	diarrheal stool	cross-sectional	5.5%	9	3
10	Rathinasamy Subashkumar	India	2006	children	216	diarrheal stool	cross-sectional	9.7%	21	7
11	M F Prère	Denmark	2006	children	280	diarrheal stool	cross-sectional	0.0%	0	5
12	A M Khan	Bangladesh	2009	children	2511	diarrheal stool	cross-sectional	8.8%	222	5
13	A Samie	South Africa	2009	children	39	diarrheal stool	cross-sectional	12.8%	5	5
14	Meiyanti	Jakarta	2010	children	117	diarrheal stool	cross-sectional	5.1%	6	1
15	M I Mota	Uruguayan	2010	children	49	diarrheal stool	cross-sectional	2.0%	1	3
16	Rathinasamy Subashkumar	India	2012	children	239	diarrheal stool	cross-sectional	30.54%	73	4
17	S Maraki	Greece	2012	children	1597	diarrheal stool	cross-sectional	0.44%	7	6
18	NASEEM Q N DUBAI	Iraq	2013	children	294	diarrheal stool	cross-sectional	4.08%	12	6
19	C Manikandan	India	2013	children	118	diarrheal stool	cross-sectional	22%	26	3
20	Manuela Onori	Italy	2014	children	245	diarrheal stool	cross-sectional	2.0%	5	3
21	M.A. Rather	India	2014	children	83	diarrheal stool	cross-sectional	7.22%	6	3
22	Sima Kazemi	Iran	2016	children	120	diarrheal stool	cross-sectional	1.7%	2	3
23	César García Vera	Spain	2016	children	729	diarrheal stool	cross-sectional	2.7%	20	7
24	Mohammad Shah	Kenya	2016	children	1410	diarrheal stool	cross-sectional	5.5%	77	7
25	Mohammad Mehdi Soltan Dallal	Iran	2016	children	391	diarrheal stool	cross-sectional	3.1%	12	7
26	Elnaz Abbasi	Iran	2016	children	200	diarrheal stool	cross-sectional	1.0%	2	6
27	Saba Talib Hashim	Iraq	2018	children	300	diarrheal stool	cross-sectional	4.0%	12	6
28	Oliver Waithaka Mbuthia	Kenya	2018	children	163	diarrheal stool	cross-sectional	4.3%	7	9
29	Sheetal Verma	India	2019	children	100	diarrheal stool	cross-sectional	1.0%	1	6
30	Mark Kilongosi Webale	Kenya	2020	children	374	diarrheal stool	cross-sectional	1.1%	4	6
31	Hamid Sadeghi	Iran	2022	children	900	diarrheal stool	cross-sectional	0.55%	5	6

### Quality assessment

Evaluation of study quality displayed that, among 31 studies, 12 had a total score of 0–4 points (Unsatisfactory Studies), 12 had a total score of 5–6 points (Satisfactory Studies), and 6 had a total score points 7–8 (Good Studies). 1 included study was considered Very Good Studies (Table 2).

**Table 2:** Newcastle Ottawa quality assessment scale of each included studies

Study	Selection				Comparability	Outcome		Total quality score
	**Representativeness of the sample**	**Sample size**	**Non-respondents**	**Ascertainment of the exposure (risk factor)**	**Confounding factors controlled**	**Assessment of outcome**	**Statistical test**	
Mustafa B Ali	*	-	-	-	-	**	-	**3**
NASEEM Q N DUBAI	*	-	*	-	**	**	-	**6**
A M Khan	*	-	-	-	**	**	-	**5**
A M Khan	*	-	-	-	**	**	*	**6**
C Manikandan	*	-	-	-	-	**	-	**3**
Manuela Onori	*	-	-	-	-	**	-	**3**
Rathinasamy Subashkumar	*	-	-	-	-	**	*	**4**
W S Lee	*	-	-	-	-	**	*	**4**
M.A. Rather	*	-	-	-	-	**	-	**3**
B Essers	*	-	*	-	**	**	-	**6**
H J Juan	*	-	-	-	-	-	-	**1**
Sima Kazemi	*	-	-	-	-	**	-	**3**
S Maraki	*	-	*	*	-	**	*	**6**
Oliver Waithaka Mbuthia	*	*	*	*	**	**	*	**9**
Meiyanti	*	-	-	-	-	-	-	**1**
M I Mota	*	-	-	-	-	**	-	**3**
Saba Talib Hashim	*	-	-	*	**	**	-	**6**
CL Obi	*	-	*	-	**	**	*	**7**
M F Prère	*	-	*	*	-	**	-	**5**
Thikra S Ali	*	-	-	*	-	**	-	**4**
A Samie	*	-	-	*	-	**	*	**5**
Mohammad Shah	*	-	*	**	-	**	*	**7**
Rathinasamy Subashkumar	*	-	*	*	**	**	-	**7**
Delfina Urbina	*	-	*	**	-	**	*	**7**
Sheetal Verma	*	-	*	*	-	**	*	**6**
Mark Kilongosi Webale	*	-	*	**	-	**	-	**6**
César García Vera	*	-	*	**	-	**	*	**7**
Mohammad Mehdi Soltan Dallal	*	-	-	*	**	**	*	**7**
H C Maltezou	*	-	-	-	-	**	*	**4**
Elnaz Abbasi	*	-	-	*	**	**	-	**6**
Hamid Sadeghi	*	-	-	*	**	**	-	**6**

### Publication bias

**Fig. 7:**
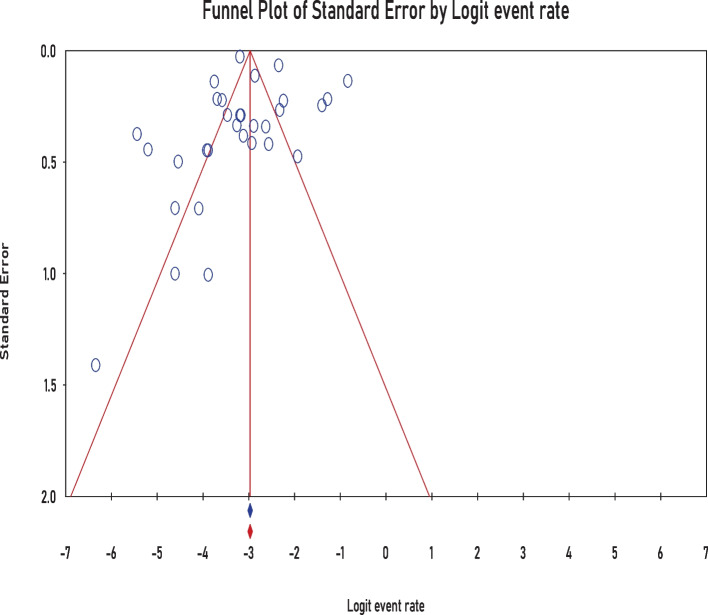
Funnel plot for the prevalence of *Aeromonas* worldwide

**Fig. 8:**
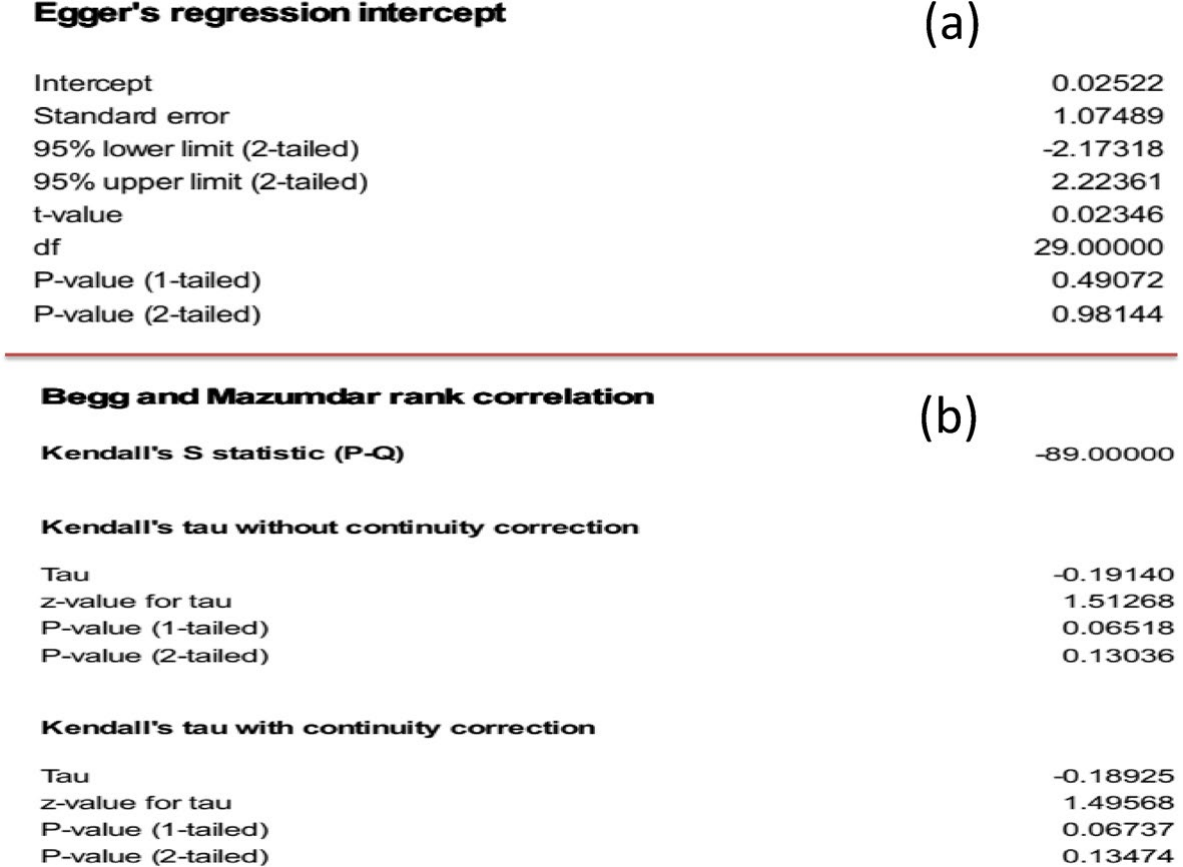
**a** Egger regression intercept for the prevalence of *Aeromonas* worldwide. **b** Begg and mazumdar rank correlation of analysis for the prevalence of *Aeromonas* worldwide

**Fig. 9:**
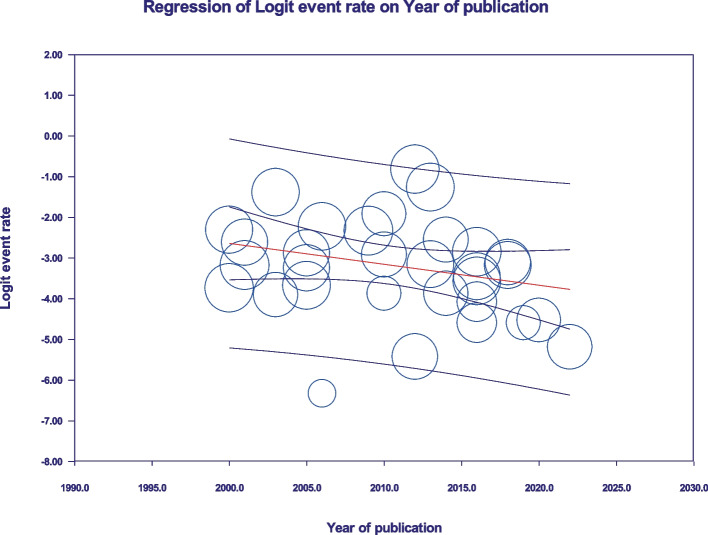
A meta-regression graph for the prevalence of *Aeromonas* in included studies based on the year of publication

As indicated by funnel plot (Fig. 7) asymmetry, no significant publication bias was observed in our study using Eggers regression intercept test (*P* = 0.98) and Begg and Mazumdar rank correlation (*P* = 0.13) (Fig. 8a, b). Meta-regression analysis revealed that there was no significant heterogeneity between studies regarding the year of publication (*P* = 0.08) (Fig. 9).

## Discussion

Enteric bacteria related diarrhoea maintains to be a main cause of morbidity and mortality among children [44]. Troeger et al. [1], *Aeromonas*-associated diarrhoeal is known as the leading cause of mortality with 1.0 cases per 100,000 which indicates that children are more sensitive to *Aeromonas* infection. In humans, *Aeromonas* is a cause of extra-intestinal and intestinal diseases, particularly in immunocompromised patients, including urinary tract infections, septicemia, wound infections, necrotizing fasciitis, and hepatobiliary tract infections [8]. Clinical symptoms of *Aeromonas* infection include chronic watery diarrhoea to severe dysentery [45]. Rehydration therapy is sufficient intervention in most children cases of gastroenteritis and watery diarrhoea caused by *Aeromonas*. Antibiotics are used for only unresponsive and sever cases of *Aeromonas* gastroenteritis or extraintestinal infections [46]. Correct detection of the genus *Aeromonas* in laboratory is still a great challenge. Many studies have been conducted with the goal of making detection applied and reproducible, thus increasing the reliability of findings [47]. The current systematic review presents the first published summary of the global prevalence of *Aeromonas* infection in children with diarrhoea. Based on 31 cross-sectional articles published during the past 22 years, we evaluated the overall prevalence of Aeromonas infection in children with diarrhoea worldwide to be 4.2% (95% CI 3.1–5.6%) using a random effect model. Global prevalence of *Aeromonas* and its huge burden in some countries such as South Africa, India, and Kenya made *Aeromonas* reportable disease especially in children with diarrhoea [37, 38, 40]. Prevalence for different studies that met the inclusion criteria of the current review differed largely from 0.002% to 30.5% [21, 35]. Differences in the prevalence of *Aeromonas* infection in children with diarrhoea reflected possible differences associated with geographic factors in various parts of the world [48, 49], water and sanitation quality, income level, and population densities [50, 51]. In the present study the greatest and lowest prevalence of *Aeromonas* infection in children with diarrhoea was in India (30.5%) [21] and Denmark (0.002%) [35]. The pooled prevalence here totally agrees with other investigations of *Aeromonas* infection in children with diarrhoea, including NASEEM Q N DUBAI (4.1%), and Oliver Waithaka Mbuthia (4.3%) [25, 52]. Furthermore, the pooled prevalence we obtained is approximately in line with the findings of both W S Lee (4.0%) and Saba Talib Hashim (4.0%) [16, 27]. Subgroups analysis based on population, income level, and water and sanitation quality score also was evaluated. Findings show prevalence of *Aeromonas* in children with diarrhoea being documented from 17 countries that have the higher prevalence in upper middle-income countries 5.1% (95% CI 2.8–9.2%), regions with populations of over 100 million people (9.4%; 95% CI 5.6–15.3%), and water and sanitation quality score of less than 25% (8.8%; 95% CI 5.2–14.4%). The present subgroups analysis confirmed the findings that the high prevalence rate of diarrhoea diseases among children would happen in areas with large population densities, poor water and sanitation facilities [50], and low-income and middle-income countries [51]. Based on the importance of quality assessment section in meta-analysis getting high-quality studies is crucial in providing reliable and useful results to provide a deeper understanding of research topic. Accordingly, some suggestions for high-quality studies are mentioned as follows:

1. High-quality research is anchored on a good study question.
2. High-quality research follows a systematic, relevant study methodology.
3. High-quality research acknowledges previous studies.
4. High-quality research uses appropriate, empirical data and correct data analysis methods.
5. High-quality research is representative and generalizable.
6. High-quality research has external validity.
7. High-quality research is replicable and transparent.

### Strengths and limitations

This was the first systematic review and meta-analysis to obtain a global prevalence of *Aeromonas* infection in children with diarrhoea. The comprehensive literature search, duplicated data elicitation, precise methodology, and quality assessment by two autonomous reviewers, obvious exclusion and inclusion criteria, and the lack of publication bias are strengths of this meta-analysis. Nevertheless, there are some limitations that goes back to the nature of the surveys that are as follow: Firstly, most of the investigations included in this meta-analysis did not report information about the virulence genes and antibiotic resistance pattern clearly and consistently; therefore, we were unable to evaluate the effect of these momentous factors. Secondly, the age of patients was not provided clearly in most included studies. Third, in many studies, information about children's gender was not given.

## Conclusion

We did provide a systematic review and meta-analysis of *Aeromonas* infection in children with diarrhoea to get a better comprehension of the global dispensation of this infectious disease. Although diarrhoeal disease fatality has reduced remarkably in the last three decades, lots of work is still required to speed up the decline in the burden of bacterial diarrhoeal diseases in deprived children in terms of safe and healthy water and sanitation, and appropriate health care.

## Data Availability

All of the data generated during and/or analyzed during the current study are available from the corresponding author on reasonable request.

## References

[1] Troeger C, Blacker BF, Khalil IA, Rao PC, Cao S, Zimsen SR (2018). Estimates of the global, regional, and national morbidity, mortality, and aetiologies of diarrhoea in 195 countries: a systematic analysis for the Global Burden of Disease Study 2016. Lancet Infect Dis.

[2] Abrami L, Fivaz M, Decroly E, Seidah NG, Jean F, Thomas G (1998). The pore-forming toxin proaerolysin is activated by furin. J Biol Chem.

[3] Guerra IM, Fadanelli R, Figueiró M, Schreiner F, Delamare APL, Wollheim C (2007). Aeromonas associated diarrhoeal disease in south Brazil: prevalence, virulence factors and antimicrobial resistance. Braz J Microbiol.

[4] Moyer NP (1987). Clinical significance of Aeromonas species isolated from patients with diarrhea. J Clin Microbiol.

[5] Albert MJ, Faruque A, Faruque S, Sack R, Mahalanabis D (1999). Case-control study of enteropathogens associated with childhood diarrhea in Dhaka, Bangladesh. J Clin Microbiol.

[6] Sadeghi H, Heidarzadeh S, Naghavi M, Rozeh ME, Afshar D (2022). Molecular detection of Aeromonas and its virulence genes in hospitalized children with diarrhea in northwest of Iran. Human Gene.

[7] Horneman AJ. Aeromonas. Manual Clin Microbiol. 2015:752–61.

[8] Janda JM, Abbott SL (2010). The genus Aeromonas: taxonomy, pathogenicity, and infection. Clin Microbiol Rev.

[9] Agger WA, McCormick J, Gurwith MJ (1985). Clinical and microbiological features of Aeromonas hydrophila-associated diarrhea. J Clin Microbiol.

[10] Liu L, Johnson HL, Cousens S, Perin J, Scott S, Lawn JE (2012). Global, regional, and national causes of child mortality: an updated systematic analysis for 2010 with time trends since 2000. Lancet.

[11] Stang A (2010). Critical evaluation of the Newcastle-Ottawa scale for the assessment of the quality of nonrandomized studies in meta-analyses. Eur J Epidemiol.

[12] Quek TT, Tam WW, Tran BX, Zhang M, Zhang Z, Ho CS (2019). The global prevalence of anxiety among medical students: a meta-analysis. Int J Environ Res Public Health.

[13] Higgins JP, Thompson SG, Deeks JJ, Altman DG (2003). Measuring inconsistency in meta-analyses. BMJ.

[14] Khan A, Faruque A, Hossain M (2005). Isolation of Vibrio cholerae from neonates admitted to an urban diarrhoeal diseases hospital in Bangladesh. Ann Trop Paediatr.

[15] Khan A, Hossain M, Khan A, Chisti M, Chowdhury F, Faruque A (2009). Bacterial enteropathogens of neonates admitted to an urban diarrhoeal hospital in Bangladesh. J Trop Pediatr.

[16] Lee W, Puthucheary S (2002). Bacterial enteropathogens isolated in childhood diarrhoea in Kuala Lumpur–the changing trend. Med J Malaysia.

[17] Maltezou H, Zafiropoulou A, Mavrikou M, Bozavoutoglou E, Liapi G, Foustoukou M (2001). Acute diarrhoea in children treated in an outpatient setting in Athens, Greece. J Infect.

[18] Manikandan C, Amsath A (2013). Antimicrobial resistance of enteric pathogens isolated from children with acute diarrhoea in Pattukkottai, Tamil Nadu, India. Int J Pure Appl Zool.

[19] Onori M, Coltella L, Mancinelli L, Argentieri M, Menichella D, Villani A (2014). Evaluation of a multiplex PCR assay for simultaneous detection of bacterial and viral enteropathogens in stool samples of paediatric patients. Diagn Microbiol Infect Dis.

[20] Rather M, Willayat M, Wani S, Munshi Z, Hussain S (2014). A multiplex PCR for detection of enterotoxin genes in Aeromonas species isolated from foods of animal origin and human diarrhoeal samples. J Appl Microbiol.

[21] Subashkumar R, Thayumanavan T, Vivekanandhan G, Lakshmanaperumalsamy P (2012). Etiology of children’s diarrhoea in Southern India: associated pathogens and usual isolates. Afr J Microbiol Res.

[22] Vera CG, Ventura MG, del Castillo AG, Aurrecoechea BD, Olcina MJE, Rubio AM (2017). Acute bacterial gastroenteritis: 729 cases recruited by a Primary Care national network. An Pediatr (Barc).

[23] Abbasi E, Khansari-Nejad B, Abtahi H, Akbari M, Ghaznavi-Rad E (2016). Low Prevalence of Aeromonas hydrophilain Infectious Diarrhea Samples of Pediatric Patients in Arak, Iran. Rep Biochem Mol Biol.

[24] Ali MB, Ghenghesh KS, Aissa RB, Abuhelfaia A, Dufani M (2005). Etiology of childhood diarrhea in Zliten, Libya. Saudi Med J.

[25] Dubai NQN, Al-Thahab AA. Isolation of outer membrane protein of Aeromonas hydrophila recoverd from children with diarrhea. Int J Humanit Arts Med Sci. 2013;1(2):15–22.

[26] Essers B, Burnens AP, Lanfranchini FM, Somaruga SG, von Vigier RO, Schaad UB (2000). Acute community-acquired diarrhea requiring hospital admission in Swiss children. Clin Infect Dis.

[27] Hashim ST, Nema MM (2018). Study of some virulence factors of Aeromonas Spp. isolated from stool samples of children with diarrhea. Iraqi J Vet Med.

[28] Juan H-J, Tang R-B, Wu T-C, Yu K-W (2000). Isolation of Aeromonas hydrophila in children with diarrhea. J Microbiol Immunol Infect.

[29] Kazemi S, Alikhani MY, Arabestani MR, Sedighi I, Rastyani S, Farhadi KH (2016). Prevalence of Aeromonas hydrophila and Yersinia enterocolitica in children with acute diarrhea attending health centers in Hamadan. Avicenna J Clin Med.

[30] Maraki S, Ladomenou F, Samonis G, Galanakis E (2012). Long-term trends in the epidemiology and resistance of childhood bacterial enteropathogens in Crete. Eur J Clin Microbiol Infect Dis.

[31] Mbuthia OW, Mathenge SG, Oyaro MO, Ng'ayo MO (2018). Etiology and pathogenicity of bacterial isolates: a cross sectional study among diarrheal children below five years in central regions of Kenya. Pan Afr Med J.

[32] Meiyanti, Salim OC, Surjawidjaja JE, Lesmana M (2010). Isolation and antibiotic sensitivity of Aeromonas from children with diarrhea. Univ Med.

[33] Mota MI, Gadea MP, Gonzalez S, Gonzalez G, Pardo L, Sirok A (2010). Bacterial pathogens associated with bloody diarrhea in Uruguayan children. Rev Argent Microbiol.

[34] Obi CL, Potgieter N, Bessong PO, Igumbor EO, Green E (2003). Prevalence of pathogenic bacteria and rotaviruses in stools of patients presenting with diarrhoea from rural communities in Venda, South Africa. S Afr J Sci.

[35] Prere MF, Bacrie SC, Baron O, Fayet O (2006). Bacterial ae tiology of diarrhoea in young children: high prevalence of enteropathogenic Escherichia coli (EPEC) not belonging to the classical EPEC serogroups. Pathol Biol (Paris).

[36] Ali TS, Dhahi SJ, Khalaf SH (2005). Study of exotoxin production ability of Aeromonas species isolated from children diarrhea in Mosul. Rafidain J Sci.

[37] Samie A, Guerrant RL, Barrett L, Bessong PO, Igumbor EO, Obi CL (2009). Prevalence of intestinal parasitic and bacterial pathogens in diarrhoeal and non-diarroeal human stools from Vhembe District, South Africa. J Health Popul Nutr.

[38] Shah M, Kathiiko C, Wada A, Odoyo E, Bundi M, Miringu G (2016). Prevalence, seasonal variation, and antibiotic resistance pattern of enteric bacterial pathogens among hospitalized diarrheic children in suburban regions of central Kenya. Trop Med Health.

[39] Soltan Dallal MM, Mazaheri Nezhad Fard R, Kavan Talkhabi M, Aghaiyan L, Salehipour Z (2016). Prevalence, virulence and antimicrobial resistance patterns of Aeromonas spp. isolated from children with diarrhea. Germs.

[40] Subashkumar R, Thayumanavan T, Vivekanandhan G, Lakshmanaperumalsamy P (2006). Occurrence of Aeromonas hydrophila in acute gasteroenteritis among children. Indian J Med Res.

[41] Urbina D, Arzuza O, Young G, Parra E, Castro R, Puello M (2003). Rotavirus type A and other enteric pathogens in stool samples from children with acute diarrhea on the Colombian northern coast. Int Microbiol.

[42] Verma S, Venkatesh V, Kumar R, Kashyap S, Kumar M, Maurya AK (2019). Etiological agents of diarrhea in hospitalized pediatric patients with special emphasis on diarrheagenic Escherichia coli in North India. J Lab Phys.

[43] Webale MK, Wanjala C, Guyah B, Shaviya N, Munyekenye GO, Nyanga PL (2020). Epidemiological patterns and antimicrobial resistance of bacterial diarrhea among children in Nairobi City, Kenya. Gastroenterol Hepatol Bed Bench.

[44] Webale MK, Wanjala C, Guyah B, Shaviya N, Munyekenye GO, Nyanga PL (2020). Epidemiological patterns and antimicrobial resistance of bacterial diarrhea among children in Nairobi City, Kenya. Gastroenterol Hepatol Bed Bench.

[45] Puthucheary SD, Puah SM, Chua KH (2012). Molecular characterization of clinical isolates of Aeromonas species from Malaysia. PLoS one.

[46] Igbinosa IH, Igumbor EU, Aghdasi F, Tom M, Okoh AI (2012). Emerging Aeromonas species infections and their significance in public health. ScientificWorldJournal.

[47] Pessoa RBG, de Oliveira WF, Marques DSC, dos Santos Correia MT, de Carvalho EVMM, Coelho LCBB (2019). The genus Aeromonas: a general approach. Microb Pathog.

[48] Hu M, Wang N, Pan Z, Lu C, Liu Y (2012). Identity and virulence properties of Aeromonas isolates from diseased fish, healthy controls and water environment in China. Lett Appl Microbiol.

[49] Ljungh A, Popoff M, Wadstrom T (1977). Aeromonas hydrophila in acute diarrheal disease: detection of enterotoxin and biotyping of strains. J Clin Microbiol.

[50] Hartley DM, Morris JG, Smith DL (2006). Hyperinfectivity: a critical element in the ability of V. cholerae to cause epidemics?. PLoS Med.

[51] Walker CLF, Rudan I, Liu L, Nair H, Theodoratou E, Bhutta ZA (2013). Global burden of childhood pneumonia and diarrhoea. Lancet.

[52] Mbuthia OW, Mathenge SG, Oyaro MO, Ng'ayo MO (2018). Etiology and pathogenicity of bacterial isolates: a cross sectional study among diarrheal children below five years in central regions of Kenya. Pan Afr Med J.

